# Amyloid-β Peptides and Tau Protein as Biomarkers in Cerebrospinal and Interstitial Fluid Following Traumatic Brain Injury: A Review of Experimental and Clinical Studies

**DOI:** 10.3389/fneur.2013.00079

**Published:** 2013-06-26

**Authors:** Parmenion P. Tsitsopoulos, Niklas Marklund

**Affiliations:** ^1^Department of Neurosurgery, Hippokratio General Hospital, Faculty of Medicine, Aristotle University, Thessaloniki, Greece; ^2^Department of Neuroscience, Division of Neurosurgery, Uppsala University, Uppsala, Sweden

**Keywords:** traumatic brain injury, biomarkers, Alzheimer’s disease, amyloid beta, tau, cerebrospinal fluid, microdialysis

## Abstract

Traumatic brain injury (TBI) survivors frequently suffer from life-long deficits in cognitive functions and a reduced quality of life. Axonal injury, observed in many severe TBI patients, results in accumulation of amyloid precursor protein (APP). Post-injury enzymatic cleavage of APP can generate amyloid-β (Aβ) peptides, a hallmark finding in Alzheimer’s disease (AD). At autopsy, brains of AD and a subset of TBI victims display some similarities including accumulation of Aβ peptides and neurofibrillary tangles of hyperphosphorylated tau proteins. Most epidemiological evidence suggests a link between TBI and AD, implying that TBI has neurodegenerative sequelae. Aβ peptides and tau may be used as biomarkers in interstitial fluid (ISF) using cerebral microdialysis and/or cerebrospinal fluid (CSF) following clinical TBI. In the present review, the available clinical and experimental literature on Aβ peptides and tau as potential biomarkers following TBI is comprehensively analyzed. Elevated CSF and ISF tau protein levels have been observed following severe TBI and suggested to correlate with clinical outcome. Although Aβ peptides are produced by normal neuronal metabolism, high levels of long and/or fibrillary Aβ peptides may be neurotoxic. Increased CSF and/or ISF Aβ levels post-injury may be related to neuronal activity and/or the presence of axonal injury. The heterogeneity of animal models, clinical cohorts, analytical techniques, and the complexity of TBI in the available studies make the clinical value of tau and Aβ as biomarkers uncertain at present. Additionally, the link between early post-injury changes in tau and Aβ peptides and the future risk of developing AD remains unclear. Future studies using methods such as rapid biomarker sampling combined with enhanced analytical techniques and/or novel pharmacological tools could provide additional information on the importance of Aβ peptides and tau protein in both the acute pathophysiology and long-term consequences of TBI.

## Introduction

In the United States, around 1.4 million people sustain a traumatic brain injury (TBI) annually (Zohar et al., 2011; Sivanandam and Thakur, 2012) and younger individuals are predominately affected (Fins, 2003; Kovesdi et al., 2010). Depending on the severity of the injury, survivors can experience significant impairments in cognition and display marked personality changes, which can have a negative impact both on the patient and the society (Magnoni and Brody, 2010; Sivanandam and Thakur, 2012). The pathophysiology of TBI is complex and involves multiple cellular and biochemical changes generated by the initial impact, leading to a disease process which exacerbate the injury for a prolonged period of time. This secondary injury process involves inflammatory cascades and heterogenous cell death pathways including apoptosis, autophagia, and necrosis (Kovesdi et al., 2007; Loane et al., 2009; Marklund and Hillered, 2011; Sivanandam and Thakur, 2012). Due to individual patient factors and initial injury characteristics, TBI produces either a focal lesion (cortical contusions, epi-subdural, or intracerebral hemorrhages), diffuse injury (diffuse axonal injury, DAI, and/or diffuse brain swelling; Strich, 1956; Yarnell and Ommaya, 1969; Gennarelli et al., 1982; Adams et al., 1989; Povlishock et al., 1992), or a mixture thereof (Saatman et al., 2008). There are substantial differences among these injury types and clinical TBI characteristics are markedly heterogeneous.

Importantly, wide-spread injury to white matter tract axons has emerged as a crucial contributor to the morbidity observed in TBI survivors (Smith and Meaney, 2000; Smith et al., 2003c; Czeiter et al., 2008). In injured axons, amyloid precursor protein (APP) accumulates mainly due to a TBI-induced disruption of axonal transport (Pierce et al., 1996). In addition, increased neuronal APP expression has also been observed in human and animal models and across the spectrum of severe TBI (Otsuka et al., 1991; Sola et al., 1993; Lewen et al., 1995; Pierce et al., 1996; Murakami et al., 1998; Ciallella et al., 2002; Itoh et al., 2009). Thus, elevated APP levels in injured axons may be due to a combination of increased neuronal expression and accumulation due to disrupted axonal transport. When APP is proteolytically cleaved by β- and γ-secretases, amyloid-β (Aβ) peptides of various lengths can be produced by normal cell metabolism and be released from the presynaptic ending of the axon in the uninjured brain (Price et al., 1995; Blennow et al., 2006; Masters et al., 2006). Experimental TBI results in increased gene and protein expression of β-secretase 1 (*BACE1*; also named β-site APP cleaving enzyme 1), the major β-secretase involved in the production of Aβ from APP in neurons (Cai et al., 2001; Blasko et al., 2004; Loane et al., 2009; Yu et al., 2012a). Although the γ-secretase presenilin-1 and BACE1 were not co-transported with APP in the sciatic nerve (Lazarov et al., 2005), BACE1 protein was found to co-accumulate with APP in injured axons following TBI in the pig (Chen et al., 2004) and in patients dying within weeks post-injury (Uryu et al., 2007). Additionally, presenilin-1 may also co-accumulate with APP in injured axons (Uryu et al., 2007). As will be discussed in the subsequent paragraphs, an association between APP accumulation and Aβ formation in injured axons, post-injury plaque deposition and the development of Alzheimer’s disease (AD) has not been firmly established. However, Aβ was found to co-accumulate with APP in injured axons up to 6 months post-injury in a miniature swine TBI model and at autopsy up to 3 years following human TBI (Chen et al., 2004, 2009). Combined, these reports argue that TBI may result in an increased production of Aβ peptides from APP. Since increased Aβ peptide generation may have neurotoxic properties and aggregate into plaques and oligomers (*vide infra*), it may have important implications in the secondary injury cascade post-TBI.

Alzheimer’s disease, the most common neurodegenerative disease, affects more than 25 million people worldwide and shows a rapidly increasing prevalence (Blennow et al., 2006). AD is primarily characterized by progressive cognitive impairments including loss of episodic memory and language, impaired judgment, decision-making, and orientation. Neuropathology is diagnostic and extracellular plaques of Aβ peptides and neurofibrillary tangles (NFTs) composed of hyperphosphorylated tau proteins are typically found in the brains of AD patients (Blennow et al., 2006; Trojanowski et al., 2010; Kennedy et al., 2012; Weiner et al., 2012). More than two decades ago, it was postulated that a single, severe TBI may result in dementia with early onset (Clinton et al., 1991). Specifically, TBI was suggested to be an independent risk factor for AD in many studies (Clinton et al., 1991; Gualtieri and Cox, 1991; Mortimer et al., 1991; Breteler et al., 1992; Mayeux et al., 1993; Guo et al., 2000). A re-analysis of 11 case control studies (Mortimer et al., 1991) and results from a cohort of 548 injured WWII veterans (Plassman et al., 2000) found that the risk for developing AD following TBI can be increased up to 4.5-fold. The association between AD and TBI was further strengthened by clinical and experimental studies demonstrating that in brain tissue from TBI survivors or from brain-injured animals, pathological findings with a resemblance to those of AD were observed (Guo et al., 2000; Johnson et al., 2010; Magnoni and Brody, 2010). A genetic factor for AD, the ε4 allele of the lipid transport apolipoprotein E (Apoε4) seems to worsen the prognosis following TBI and predispose to the formation of Aβ plaques in AD (Nicoll et al., 1995; Kim et al., 2009). These reports argue that TBI may be a risk factor for the long-term development of AD (Mortimer et al., 1991; Plassman et al., 2000; Fleminger et al., 2003; Johnson et al., 2010; Magnoni and Brody, 2010).

Despite this suggested link between TBI and AD, numerous unanswered questions remain. For instance, is the increased risk of AD after TBI a direct consequence of cascades initiated at the time of impact, reflected by initial changes in Aβ and tau levels in brain, cerebrospinal fluid (CSF), and/or interstitial fluid (ISF)? Alternatively, does the TBI-AD link merely reflect a hastened cognitive decline and/or a reduced cognitive reserve induced by TBI? Specifically, in recent *in vivo* studies, Aβ and/or tau have been analyzed as biomarkers in both the experimental and clinical TBI setting in the CSF or in the ISF using microdialysis (MD). Although the analysis of phospho-tau and Aβ peptides is crucial in the diagnosis of AD (Mattsson et al., 2012), the interpretation of tau and Aβ peptides following TBI is unclear. Compared to most AD models, the data on Aβ and tau formation following experimental TBI are, to some extent, highly heterogeneous and AD pathology has not been robustly confirmed. In fact, rodent TBI models have been unable to show the hallmark findings of NFTs and Aβ plaques post-TBI. Regardless, since tau and Aβ levels may markedly influence the pathophysiology of TBI, both acutely and at long-term, they can potentially be used as biomarkers. In this review, we focus on the available evidence for increased Aβ and tau pathology in injured brain tissue and the use of Aβ peptides and tau as potential biomarkers in the CSF and ISF following TBI.

## Aβ and Tau Histopathology Following TBI-Animal Studies

Due to the heterogeneity of clinical TBI, numerous animal models exist (Marklund and Hillered, 2011). To date, most TBI studies evaluating tau and Aβ have used the focal controlled cortical impact (CCI) model, and only infrequently have models of diffuse TBI producing wide-spread axonal injury been evaluated (Tables 1 and 2). In initial TBI studies in rats, immunohistochemical analysis (IHC) revealed accumulation of APP in injured axons although Aβ peptides were not detected (Lewen et al., 1995; Pierce et al., 1996). Instead, mice overexpressing human APP [APP-yeast artificial chromosome (APP-YAC mice), PDAPP, and recently 3xTg-AD mice] displaying Aβ plaque pathology were developed and studied using the CCI model (Murai et al., 1998; Nakagawa et al., 1999, 2000; Hartman et al., 2002; Uryu et al., 2002; Conte et al., 2004; Tran et al., 2011a, 2012) (Table 1). Non-transgenic mice “knocked-in” with the human Aβ coding sequence to their endogenous APP gene (APP^NLh/NLh^) have also been developed (Abrahamson et al., 2006, 2009). Although these models failed to mimic the formation of Aβ plaques similar to that observed in humans, findings such as exacerbated cell death and brain atrophy in APP-overexpressing mice were noted post-TBI (Smith et al., 1998). Since a *decreased* plaque load was found in aged plaque-forming PDAPP transgenic mice following TBI, plaque pathology may be potentially reversible (Nakagawa et al., 2000).

**Table 1:** **Animal studies on traumatic brain injury (TBI) and Aβ**.

Reference	Type of animal	Age	Animal model	Aβ detection technique	Aβ peptide	Time post-injury	Plaque formation	Major findings
Murai et al. (1998)	APP ↑ ↑mice	12–15 m	CCI	IHC, ELISA	↓Aβx-40, Aβx-42/43 ↔	1 w	No	APP-overexpressing mice were unaltered in lesion volume and behavior. Punctate cortical Aβ-IR ↑by TBI
Smith et al. (1998)	PDAPP mice	4 m	CCI	IHC, ELISA	Sevenfold ↑Aβ40, threefold ↑Aβ42	2 h	No increase by TBI	In PDAPP mice, both Aβ40 and Aβ42 levels were increased early post-TBI associated with ↓cognition and ↑neuronal cell death
Nakagawa et al. (1999)	PDAPP mice	4 m	CCI	IHC	↓Aβx-40, Aβx-42	5–8 m	No	Exacerbated hippocampal atrophy in PDAPP mice post-injury. TBI reduced Aβx-40 and Aβx-42 burden in the transgenic mice
Nakagawa et al. (2000)	PDAPP mice	24 m	CCI	IHC	↓Aβ	1–16 w	Reduction by TBI in PDAPP mice	Hippocampal atrophy worse after TBI in PDAPP mice. Aβ plaque burden reduced by TBI
Hartman et al. (2002)	PDAPP ±human APOE4	8–9 m	CCI	IHC	↑Aβ-ip	3 m	In PDAPP/APOE4+ mice only	AP deposits in PDAPP mice (diffuse plaques). TBI accelerates Aβ deposition in PDAPP mice in Apoe4 presence neuron loss ↔
Uryu et al. (2002)	Tg2576 and wild-type mice	9 m	CCI	IHC, ELISA	↑Aβ	9–16 w	No	Aβ burden mildly increased in both single and repetitive mTBI mice compared to sham-injured controls
Conte et al. (2004)	Tg2576 mice	11 m	CCI	IHC	↑Aβ40 and Aβ42	8 w	No	Vitamin E attenuated learning deficit and TBI-induced Aβ increases following repetitive mild TBI
Abrahamson et al. (2006)	APP^N*Lh* N*Lh*^ mice	3 m	CCI	IHC, ELISA, WB	↑Aβ40, ↑ ↑Aβ42	3 h–14 d	No	A caspase inhibitor attenuated the TBI-induced increase in APP and Aβ and improved histological outcome
Abrahamson et al. (2009)	APP^N*Lh* N*Lh*^ mice	3 m	CCI	ELISA	↑Aβ40, Aβ42 ∼two to threefold increase	3–7 d	No	Simvastatin attenuated TBI-induced increases in hippocampal Aβ levels and improved behavioral outcome
Loane et al. (2009)	BACE1 KO mice	11–12 m	CCI	ELISA	↑Aβx-40	1–7 d	No	Genetic (β-) of pharmacological (γ-) inhibition of secretases improved motor, cognitive, and histological outcome
Tran et al. (2011b, 2012)	3xTg-AD mice	5–7 m	CCI	IHC, ELISA, WB	↑Aβ, Aβ40	1–24 h	No	Intra-axonal Aβ accumulation early and increased Aβ in ipsilateral hippocampus
Mannix et al. (2011)	BACE1 KO mice	2–3 m	CCI	ELISA	↑Aβ1-40	23 d	No	Young BACE1 KO had lower Aβ1-40 pre- and post-injury and markedly impaired behavioral outcome
Yu et al. (2012b)	WT mice	7 w	CCI	IHC, ELISA, WB	Aβ oligomers Aβ42	3 d	No	Levels of Aβ42 and Aβ oligomers were found to be significantly increased in the hippocampus after TBI. Lithium attenuated TBI-induced Aβ load and functional deficits
Schwetye et al. (2010)	PDAPP, Tg2576 mice	3–6 m	CCI	MD, ELISA	Baseline Aβ1-x ↑in transgenics, ↓Aβ1-x after TBI	2–24 h	No	Aβ levels in interstitial fluid were immediately decreased by 25–50% in the ipsilateral hippocampus following TBI
Smith et al. (1999)	Miniature swine	4 m	RA	IHC	↑Aβ	3–10 d	Diffuse plaques in 1/3	Accumulation of Aβ and tau together with, e.g., APP in injured axons. Few plaques in white matter tracts and layer III in cortex
Iwata et al. (2002)	Rats	3–4 m	LFP	IHC, WB, ISH	↑Aβ	1 m–1 y	No	Accumulation of Aβ and strong immunoreactivity in injured axons
Stone et al. (2002)	Rats	N/A	I/A	IHC	↑Aβ	48 h	No	Aβ formation in foci of axonal injury
Chen et al. (2004)	Miniature swine	6 m	RA	IHC, WB, ELISA	↑Aβ, APP	3 d–6 m	Yes, in gray and white matter	Co-accumulation APP and Aβ peptide in injured axons
Tian et al. (2012)	Rats	N/A	WD	IHC, ELISA, WB	↑Aβ42	14 d	No	TBI increased Aβ42 expression-Aβ42 deposits attenuated by intranasal NGF

*In the vast majority of rodent studies, the CCI model of focal TBI was used and only rarely were TBI models with a higher degree of axonal injury evaluated. The age of the animals, genetic profile and modification, time post-injury, and analytical techniques may all have contributed to the inconclusive or negative findings in many of these studies. Amyloid plaque formation was consistently observed in pig models although displaying smaller and more diffuse plaques compared to human TBI. Thus, currently available animal models may not perfectly mimic the plaque-forming capacity observed in a subset of TBI patients.APP, amyloid precursor protein; BACE1, beta-secretase 1; CCI, controlled cortical impact; d, post-injury day; ELISA, enzyme-linked immunosorbent assay; F, female; HC, hippocampus; I/A, impact-acceleration; IHC, immunohistochemistry; IR, immunoreactivity; ISH, in situ hybridization; LFP, lateral fluid percussion; M, male; MD, microdialysis; m, months; N/A, data not available; NGF, nerve growth factor; PDAPP, platelet-derived amyloid-beta precursor protein; RA, rotational acceleration; WB, western blotting; WD, weight drop; wt, weight. All rats in this Table are Sprague–Dawleys*.

**Table 2:** **Animal studies on traumatic brain injury (TBI) and tau**.

Reference	Animal	Age	Animal model	Tau detection technique	Tau type	Time detected	Major findings
Hoshino et al. (1998)	Rat	3 m	LFP	IHC	P-tau	6 m	Six months after TBI, numerous neurons were immunoreactive for P-tau or Aβ
Smith et al. (1999)	Pig	4 m	RA	IHC	T-tau	3–10 d	Accumulations of T-tau and NF-rich inclusions were found in neuronal perikarya. Tau accumulated in most axonal bulbs
Liliang et al. (2010a)	Rat	N/A	WD	ELISA, WB	T-tau	1–6 h	T-Tau levels ↑ ↑at 1 h, returned to baseline by 6 h post-injury. Tau levels were higher in the severe TBI group compared to the mild TBI group
Genis et al. (2000)	ApoE-deficient mice	4 m	WD CHI	WB	P-tau, T-tau	4–24 h	P-tau increased at baseline in transgenics. In WT controls, P-tau ↑at 4 h post-TBI, returned to baseline at 24 h. Minimal increase in P-tau in transgenics, clearly less than in WT controls
Yoshiyama et al. (2005)	T44tauTg and WT non-Tg mice	12 m	Mild repetitive	BC, IHC, WB	NFT*	9 m	Behavioral outcome not impaired 6 months post-TBI. Only one Tg T44 mouse only showed extensive NFTs and cerebral atrophy
Gabbita et al. (2005)	Adult rat^1^	Adult	CCI	ELISA, IB	C-tau	6–168 h	C-tau levels was increased at 6 h post-TBI, peaked at 168 h post-injury. Elevated brain C-tau levels associated with TBI-induced tissue loss
Tran et al. (2011a)	3xTg-AD and wild-type B6/SJL mice	5–7 m	CCI	ELISA, IHC, WB	P-tau	24 h–7 d	In 3xTg-AD mice, TBI resulted in increased intra-axonal phospho-tau immunoreactivity after TBI
Tran et al. (2011b)	3xTg-AD, APP/PS1, TauP301L mice	2–6 m	CCI	IHC, WB	T-tau, P-tau	1–24 h	Increased tau pathology early in 3xTg-AD and TauP301L mice with a peak at 1 and 24 h post-TBI. Increase in contralateral hippocampus beginning at 12 h post-TBI. P-tau increased in fimbriae and fornix
Tran et al. (2012)	3xTg-AD mice	5–7 m	CCI	WB, IHC, HP	P-tau	24 h	Abnormal co-accumulation of several phosphorylating kinases with tau at 24 h post-TBI. A JNK inhibitor reduced P-tau accumulation in axons
Yu et al. (2012b)	WT mice	7 w	CCI	IHC, WB, ELISA	P-tau	3 d	P-Tau was increased in the thalamus post-TBI; lithium administration reduced P-tau at 3 d post-TBI, resulting in improved spatial learning
Ojo et al. (2013)	h-Tau mice	18 m	Repetitive mTBI	IHC	P-tau	21 d	Increased P-tau by repetitive, 48 h apart, mTBI although not a single mTBI

In both focal and diffuse TBI models did the levels and expression of tau consistently increase post-injury.APP, amyloid precursor protein; BC, biochemical analysis; CCI, controlled cortical impact; C-tau, cleaved-tau; ELISA, enzyme-linked immunosorbent assay; HP, histopathological analysis; h-tau, mice overexpressing human tau; IB, immunoblotting; IHC, immunohistochemistry; LFP, lateral fluid percussion; m, month; P-tau, phosphorylated tau; PS1, presenilin-1; JNK, c-Jun N-terminal kinase; RA, rotational acceleration; T-tau, total tau; N/A, data not available; NFT, neurofibrillary tangles; TBI, traumatic brain injury; w, week; WB, western blotting; WT, wild-type; CHI, closed head injury; WD, weight drop; mTBI, mild traumatic brain injury; ^1^Age not mentioned; *NFTs were observed in one mouse only suggesting this study was negative for producing a tauopathy post-injury.

When rats were evaluated in the impact/acceleration and lateral fluid percussion injury models, both showing wide-spread axonal injury, long-term accumulation of Aβ in injured axons was noted although not Aβ plaques (Iwata et al., 2002; Stone et al., 2002; Tian et al., 2012). Although recent studies using various Enzyme-Linked Immunosorbent Assay (ELISA) and immunohistochemical detection methods have shown increased Aβ load in wild-type animals (Loane et al., 2009; Mannix et al., 2011; Tian et al., 2012), the vast majority of rodent TBI mice models failed to replicate the Aβ plaque formation observed in humans (see Table 1). To date, only in PDAPP-human APOε4 transgenic mice was TBI found to accelerate Aβ plaque formation (Hartman et al., 2002). Since the rodent Aβ sequence differs from the one in humans at amino acid positions 5, 10, and 13 (Selkoe, 1989), poor immunohistochemical detection techniques and less aggregating properties of mouse Aβ was suggested (Smith et al., 1998). At present, improved immunohistochemical methods have alleviated this problem of Aβ detection in rodents and additionally, APP transgenic animals carry the human sequence. The increased Aβ load noted in some animal TBI models may be dependent on the evaluated Aβ species, time span post-injury, and age of the animal. It is also plausible that Aβ formation is more extensive in TBI models with a higher degree of axonal injury.

Although rodent TBI models produce pathology similar to that observed in humans, there are obvious differences in anatomy as well as gray-white matter ratio, and rodents are also lissencephalic (Morales et al., 2005; Marklund and Hillered, 2011). Thus, high-order species may have advantages in terms of clinical relevance and AD-like pathology was evaluated in a rotational acceleration DAI model in miniature swine (Meaney et al., 1995; Smith et al., 1997; Johnson et al., 2010). Although a smaller number of Aβ plaques compared to TBI patients was observed, this model produced Aβ accumulation in injured axons in addition to plaque formation (Smith et al., 1999; Chen et al., 2004). Furthermore, diffuse Aβ plaques in both gray and white matter were identified (Smith et al., 1999) and APP co-accumulated with Aβ post-injury (Chen et al., 2004) (Table 1). This swine model appears suitable for the study of Aβ pathology following TBI, particularly in relation to axonal injury.

The microtubule-associated protein tau has six isoforms in humans and is a normal constituent primarily of axons. In pathological conditions such as TBI, tau can be hyperphosphorylated (P-tau) and aggregate which is needed for the formation of NFTs (Geddes et al., 1999; McKee et al., 2009; Ojo et al., 2013). Tau dissociated from microtubuli can disperse not only by interneuronal transfer but also via glial to glial spread (Genis et al., 2000; Tran et al., 2011a,b), be involved in Aβ-induced neurotoxicity (Rapoport et al., 2002) and also be neurotoxic by itself (Farias et al., 2011). The formation of NFTs has been observed both following repetitive mild human TBI and many years following a single severe TBI in a subset of patients in addition to its crucial role in AD. Tau formation has been evaluated in numerous experimental TBI studies using Western Blot, ELISAs, and immunohistochemistry (Hoshino et al., 1998; Smith et al., 1999; Genis et al., 2000; Ikonomovic et al., 2004; Gabbita et al., 2005; Yoshiyama et al., 2005; Uryu et al., 2007; Liliang et al., 2010a,b; Tran et al., 2011a,b, 2012; Rostami et al., 2012; Yu et al., 2012b; Ojo et al., 2013). The vast majority of rodent studies have used focal TBI models and evaluated changes in total tau (T-tau), cleaved-tau (C-tau), and/or P-tau within the first post-injury weeks (Table 2). Importantly, these rodent models have not been able to reproduce the NFT pathology observed in AD.

Several studies have used transgenic mice in the study of tau pathology following TBI (Genis et al., 2000; Yoshiyama et al., 2005; Tran et al., 2011a,b, 2012; Yu et al., 2012b). Although both wild-type and Apoε-deficient mice showed tau hyperphosphorylation post-injury following closed head injury, it was more marked in wild-type controls (Genis et al., 2000). These important findings need to be reproduced also in other TBI models. Importantly, accumulation of phosphorylated tau over time may influence neuronal structure and synaptic properties (Dickstein et al., 2010). Due to the increasing interest in the long-term sequelae of mild repetitive TBI in humans (e.g., concussions in sports), repeated mild TBIs in mice have been evaluated. Although NFTs or behavioral deficits were not induced by repeated mTBI in transgenic mice expressing the shortest human tau isoform (Yoshiyama et al., 2005), increased P-tau without NFT formation was observed following repeated mTBI in aged mice overexpressing human tau (Ojo et al., 2013).

In summary, the swine, wild-type rodents, and transgenic mice TBI models thus consistently showed increased tau protein levels post-injury without producing the NFTs observed in AD. Importantly, most animal TBI studies negative for NFT formation have only used short-term survival whereas NFT was only observed in patients surviving for many years following severe TBI although not in patients dying within 4 weeks of the injury (Smith et al., 2003a; Johnson et al., 2012).

## Aβ and Tau Histopathology Following TBI-Human Studies

In approximately 30% of patients dying early from TBI, Aβ plaques was identified at autopsy across all age groups (Gentleman et al., 1993, 1997; Roberts et al., 1994; Horsburgh et al., 2000; Smith et al., 2003b; Uryu et al., 2007; Chen et al., 2009). Diffuse Aβ plaques have been also observed by immunohistochemistry in surgically removed focal injuries within days post-injury (Ikonomovic et al., 2004; DeKosky et al., 2007). Aβ plaques have also been found in injured axons of DAI patients dying<9 days post-injury (Smith et al., 2003c). Importantly, wide-spread Aβ pathology can remain for many years in the brains of survivors of moderate to severe TBI (Johnson et al., 2010, 2012). Contrary to the diffuse plaques observed acutely, these long-term Aβ plaques were more often fibrillary (Johnson et al., 2012). Since Aβ plaques are found in only ∼30% of TBI patients, the development of neurodegeneration and/or AD likely has a multifactorial basis including altered expression of, e.g., the Aβ-degrading enzyme neprilysin gene which is related with some forms of AD (Helisalmi et al., 2004). Notably, neprilysin gene polymorphism was linked to the occurrence of Aβ plaques following TBI (Johnson et al., 2009), raising the possibility to screen individuals with a high risk of TBI such as participants in contact sports or soldiers.

Numerous clinical reports have reported tau pathology, in particular an accumulation of NFTs, in the brains of athletes who sustained several concussions during their career. This entity has been named chronic traumatic encephalopathy (CTE) (Corsellis et al., 1973; Roberts et al., 1990; Dale et al., 1991; Geddes et al., 1999; McKee et al., 2009, 2013). Although these findings have also been observed in athletes from a variety of different sports including American football or ice hockey, they have been classically seen in the brains of up to 17% of former professional boxers (previously named *dementia pugilistica* or “punch-drunk” syndrome) (Roberts et al., 1990). Common symptoms in CTE include memory loss, Parkinson-like movements, and dementia (Roberts et al., 1990; Jordan et al., 1995; McKenzie et al., 1996; McKee et al., 2009; Nowak et al., 2009). In CTE, the vast majority of cases display wide-spread NFTs and Aβ pathology is much less frequently observed (McKee et al., 2013). Recently, the largest cohort of individuals to date with a history of repeated concussions was analyzed where wide-spread tauopathy was observed (McKee et al., 2013). Although these reports and others suggest that repeated concussions/mTBI should be regarded very seriously, the number of examined individuals is still low and the incidence of CTE, its risk factors, and the contribution of other co-variables has yet to be defined.

Tau pathology, including high density and wide-spread NFTs, was also observed in patients who suffered a single, severe TBI 1–47 years previously (Johnson et al., 2012). In this study, 39 patients with a single, severe TBI surviving for more than 1 year post-injury were compared to 47 age-matched controls. Mean survival was 8 years and NFTs were present in 34% of patients<60 years old compared to 9% of controls of similar age. Additionally, the NFTs in TBI patients were commonly observed in superficial cortical layers, in depths of the sulci, and clusters were observed in the cingulate gyrus, the insular cortex, and the superior frontal gyrus. In contrast, NFTs were rarely observed outside the transentorhinal cortex and the CA1 in controls (Johnson et al., 2012). This study was the first to observe NFT at long-term following a single, severe TBI in humans although additional studies including a larger number of patients are required for confirmation of these findings. The long delay between the injury and the NFT analysis and the large age span in this patient cohort add to the inherent variability and many potential co-variables may have contributed to the formation of NFTs (Johnson et al., 2012). The process of delayed NFT formation in human TBI, if at all present, remains to be defined. Early following severe TBI, total and phospho-tau protein was found to accumulate in both neuronal cell bodies and axons post-TBI in a subset of patients (Smith et al., 2003a; Uryu et al., 2007) although without clear NFT pathology. In surgically resected brain tissue early post-injury, diffuse neuronal tau immunostaining was observed in most patients, although only 2/18 patients showed NFTs (Ikonomovic et al., 2004). In addition, NFTs were not found in TBI patients who died within 4 weeks from injury (Smith et al., 2003a), suggesting that the mechanisms leading to NFT and/or CTE pathology requires a prolonged time post-injury to develop.

Thus, numerous animal and human observations support a link between AD and TBI. However, there are substantial clinical and histopathological differences between AD and TBI (Johnson et al., 2010). In the brains of CTE victims, P-tau immunoreactive NFTs are found superficially in wide-spread cortical regions (Hof et al., 1992; McKee et al., 2009) in contrast to AD where NFT are predominately observed in deep cortical layers. Additionally, typical neuritic plaques with a dense core of fibrillar Aβ represent a typical finding in AD patients, whereas diffuse Aβ plaques with non-fibrillary Aβ are observed early in TBI (Horsburgh et al., 2000; Johnson et al., 2010). The Aβ plaques observed in AD develop over several years and are typically seen in older individuals in contrast to TBI, where Aβ plaques have been demonstrated as early as 2 h post-injury and in young patients as well (Roberts et al., 1994; Ikonomovic et al., 2004; Johnson et al., 2010). Additionally, TBI Aβ plaques appear more in the gray matter in contrast to AD (Smith et al., 2003b) and it is unclear whether the diffuse TBI-induced Aβ plaques progress into the more solid and dense plaques characteristic of advanced AD (Horsburgh et al., 2000; Chen et al., 2009; Johnson et al., 2010, 2012). Several years following a single, severe TBI, fibrillary Aβ plaques have been observed, implying that TBI may accelerate the pathophysiological process leading to AD. These data suggest that the mechanisms leading to an increased risk for neurodegeneration and AD following TBI are highly complex.

## Rationale of Aβ Peptides as Biomarkers Following TBI

*In vitro* and animal AD models indicate that Aβ accumulation, in particular the soluble oligomeric form, may be a crucial initiating factor in AD (LaFerla et al., 2007; Gouras et al., 2010) preceding tau-related neurotoxicity (Hardy and Selkoe, 2002). However, both *in vitro* and *in vivo* animal studies demonstrate that extracellular Aβ concentrations are regulated by neuronal metabolism and synaptic activity (Cirrito et al., 2005, 2008). The majority (80–90%) of generated Aβ peptides consist of the 40-amino acid long peptide Aβ1-40 (Aβ40). The longer Aβ1-42 (Aβ42) proteolytic variant is more hydrophobic and tends to aggregate into plaques (Brody et al., 2008). In the experimental setting, Aβ may be synaptotoxic (Claeysen et al., 2012; Koffie et al., 2012), neurotoxic (Walsh et al., 2002), disrupt cellular membranes (Berman et al., 2008), interfere with mitochondrial function (Parihar and Brewer, 2010), activate NMDA receptors (Texido et al., 2011), or activate microglia (Stalder et al., 1999). Importantly, both endogenously and exogenously elevated Aβ may lead to neuronal death and behavioral dysfunction (Mattson, 2004). Since Aβ peptides co-accumulate with APP (Smith et al., 1999, 2003b; Uryu et al., 2007), damaged axons may be a key source of Aβ, released into the surrounding tissue due to lysis or leakage (Smith et al., 2003c).

Therefore, since neuronal/axonal Aβ peptides, released from normal neuronal activity and/or from increased production via injury-induced accumulation of APP, are implicated in the secondary injury process, Aβ peptides sampled from CSF (Table 3) or ISF (Table 4) are of interest as biomarkers in TBI.

**Table 3:** **Amyloid β and tau levels in cerebrospinal fluid (CSF) in patients with traumatic brain injury**.

Reference	Patients (*N*)	Age (years)	Sample period	Aβ1-40		Aβ1-42		Tau	
	
	
	
				TBI	Control	TBI	Control	TBI	Control
^a^Raby et al. (1998), Emmerling et al. (2000)	6, severe TBI	35.5 (19–51)	3 w	0.94 ± 0.08 ng/mg	1.59 ± 0.53 ng/mg	1.17 ± 0.11 ng/mg	0.38 ± 0.2 ng/mg	2308 ng/ml	N/A
^b^Zemlan et al. (1999)	15, severe TBI	32.4 ± 14.1	1–8 dpi	N/A	N/A	N/A	N/A	C-tau: 1519 ± 3019 pg/ml	C-tau: 0–31 pg/ml
^b^Zemlan et al. (2002)	28, severe TBI	35.1 (18–75)	1–7 dpi	N/A	N/A	N/A	N/A	C-tau ventricular: d 1: 3205 pg/ml d 3: 556 pg/ml	C-tau lumbar: 75 ± 86 pg/ml
^c^Franz et al. (2003)	29, severe TBI *n* = 15 vCSF *n* = 14 lCSF	41 (15–72)	1–284 dpi	N/A	N/A	167 (120–477) pg/ml	284 (172–564) pg/ml; 388 (256–768) pg/ml	1756 (35–5720) pg/ml	193 (16–326) pg/ml^1^; 109 (69–159) pg/ml^1^
^d^Olsson et al. (2004)	28, severe TBI	41 (15–81)	0–11 dpi	N/A	N/A	96 (79–196) pg/ml (d 7–11)	N/A	N/A	N/A
^d^Ost et al. (2006)	39, severe TBI	49 (16–82)	0–14 dpi	N/A	N/A	N/A	N/A	T-tau (d 2–3): 682 and IQR 1155 pg/ml and 8500 and IQR 7630 pg/ml^2^	T-tau: 677 and IQR 308 pg/ml
^b^Zetterberg et al. (2006)	14, boxers	22 ± 3.8	7 dpi–3 m	19400 ± 50 ng/L	19300 ± 2740 ng/L	858 ± 128 ng/L	773 ± 133 ng/L	T-tau: 449 ± 176 ng/L; P-tau: 37.9 ± 10.2 ng/L	T-tau: 325 ± 97.7 ng/L; P-tau: 46.4 ± 14.5 ng/L
^a^Neselius et al. (2012)	30, boxers	22 (17–34)	^a^1–6 dpi; ^b^>14 dpi	N/A	N/A	^a^306 ± 52 ng/L; ^b^294 ± 54 ng/L	297 ± 039 ng/L	^a^T-tau: 58 ± 25 ng/L; ^b^T-tau: 49 ± 21 ng/L	T-tau: 45 ± 17 ng/L

*Both Aβ and tau levels following TBI show a large variability due, e.g., to the heterogeneity of the study protocols, patient cohorts, analytical techniques, and post-injury time point.^1^Control values included patients with dementia and headache; ^2^survivors versus non-survivors; dpi, day post-injury; NPH, normal pressure hydrocephalus; l, lumbar; N/A, not available; v, ventricular; CSF, cerebrospinal fluid. Data presented as ^*a*^Means and range; ^*b*^means ± Standard Deviations; ^*c*^medians and range; ^*d*^medians and interquartile range (IQR). ELISA was used to determine Aβ levels in all studies*.

**Table 4:** **Amyloid β and tau levels in interstitial fluid (ISF) in patients with traumatic brain injury-microdialysis (MD) studies**.

Reference	Patients (*N*)	Type of injury	Catheter location	Catheter	Sample interval	Analyte	Analysis method	ISF levels (pg/ml)	ISF tau levels (pg/ml)	Major findings
Brody et al. (2008)	19	Severe TBI (*n* = 12); SAH (*n* = 6); unruptured aneurysm (*n* = 1)	Frontal in most patients	CMA70 (*n* = 6) CMA71 (*n* = 13)	Aβ1-x:1, every 2 h; Aβ 1–40 and Aβ 1–42, every 8 h	Aβ1-x Aβ 1–40 Aβ 1–42	ELISA	Not reported; estimated from Figures: Aβ42; most MD samples between 10 and 60 pg/ml Aβ1-x: median 1000 pg/ml	N/A	A positive correlation between changes in brain interstitial fluid Aβ concentration and neurological status was found
Marklund et al. (2009)	8	Severe TBI, focal/mixed (*n* = 5), DAI (*n* = 3)	Frontal (*n* = 6); peri-C (*n* = 2)	CMA71	Every 1 h	Aβ40, Aβ42, T-tau	ELISA	Aβ42 (median and range): 167 pg/ml (31–295)	T-tau: 2881 ± 1774 pg/ml (121–6500) Means ± SD and range	High levels of Aβ42 in ISF post-injury. Aβ42 levels were higher in DAI patients. Tau protein levels were higher in patients with focal/mixed disease
							
Magnoni et al. (2012)	16	DAI (*n* = 8), EML (*n* = 7), nEML (*n* = 1)	Frontal (*n* = 10); peri-C (n = 6)	CMA71	Every 1–2 h, every 4–6 h for most patients	Aβ1-x, T-tau, NF-L	ELISA	First 24 h (median and range): peri-C Aβ1-x: 270 pg/ml (83–417); non-C Aβ: 1023 pg/ml (778–1968)	First 24 h: peri-C T-tau: 15950 pg/ml (11390–27240); non-C T-tau: 3469 pg/ml (1684–8691)^1^	Patients in the pericontusional group had lower Aβ and higher tau levels compared to patients in the non-contusional group. Initial tau levels were inversely correlated with initial Aβ levels. *In vitro* recovery for αβ was 30 and 1–2% for tau

*Since the normal, injured tau, and Aβ peptide levels in the injured human brain are unknown it is yet difficult to establish the magnitude of TBI-induced alterations. Both increased and decreased Aβ peptide levels have been suggested depending on injury site and catheter location. Aβ peptide levels may be increased due to their formation in injured axons and also be related to the level of consciousness and degree of neuronal activity. Interstitial tau levels may be higher in patients with a focal disease and be inversely correlated with Aβ peptide levels. It appears that MD is a useful tool for the study of Aβ and tau dynamics in the injured human brain following TBI.Aβ, beta amyloid; DAI, diffuse axonal injury; ELISA, enzyme-linked immunosorbent assay; EML, evacuated mass lesion; nEML, non-evacuated mass lesion, MD, microdialysis; N/A, not available; NF-L, neurofilament light chain; Non-C, non-contusional; Peri-C, pericontusional; SAH, subarachnoidal hemorrhage; TBI, traumatic brain injury; SD, standard deviations*.

## CSF Biomarkers of Aβ Pathology Following TBI

In the human CSF, Aβ peptides are found throughout life in their soluble forms. Studies of AD patients have shown that low CSF Aβ42 concentrations correlate with a high number of brain plaques (Strozyk et al., 2003). Additionally, some studies have found increased diagnostic accuracy of the Aβ42/Aβ40 ratio compared to Aβ42 alone (Hansson et al., 2007).

When the antibodies R165, which specifically recognize Aβ42 and R163, reacting only with Aβ40, were used in combination with Western Blot and ELISA, CSF Aβ40 and Aβ42 levels were found to be increased early following severe TBI (Raby et al., 1998; Emmerling et al., 2000) in contrast to normal, (∼50 pg/ml), plasma levels. On the contrary, decreased CSF Aβ40 and Aβ42 concentrations have also been observed (Franz et al., 2003; Kay et al., 2003) and associated with poor clinical outcome (Franz et al., 2003). In lumbar CSF, the Aβ40, Aβ42, and total Aβ levels are highly correlated and may fluctuate markedly over time when serial taps are used (Bateman et al., 2007). Similar studies in TBI, where CSF samples are frequently obtained from ventricular CSF, are lacking.

The driving force of Aβ peptides from brain parenchyma into the interstitial and intraventricular compartments are yet incompletely understood following TBI and may be related to the presence of cerebral edema and the function of the blood-brain and brain-CSF barriers (Brightman and Kaya, 2000; Iliff et al., 2012). The CSF levels of Aβ40 and Aβ42 in controls and AD patients differ markedly among published studies (Mehta et al., 2000; Frankfort et al., 2008), similar to the observations in the available TBI studies (Table 3). Thus, it is plausible that the evaluation method, time post-injury and TBI severity, Apoε4 and neprilysin gene status, the presence of TBI-induced Aβ plaques, and yet undetermined factors may all influence Aβ levels in CSF. Future studies combining CSF with ISF levels correlating tissue and behavioral outcome in addition to the analysis of yet other Aβ peptide species are needed to determine the clinical value of CSF Aβ peptide levels as biomarkers.

## Interstitial Fluid Biomarkers of Aβ Pathology Following TBI

Microdialysis sampling of the ISF has been used for more than two decades for neurochemical monitoring of the human brain (Hillered and Persson, 1999; Bellander et al., 2004; Hillered et al., 2005). MD may be considered mainly a focal sampling method in contrast to CSF sampling, which reflects more global events (Hillered et al., 2005). Aβ peptides are regarded normal constituents of human ISF (Seubert et al., 1992), possibly reflecting a physiological secretion from neuronal metabolism (Hong et al., 2011). In the pathogenesis of AD, Aβ can aggregate into insoluble species and Aβ oligomeric forms, which have been shown to be cytotoxic and influence synaptic function (Funke, 2011; Hard, 2011). Although initial Aβ aggregation can occur intracellularly and/or extracellularly (Meyer-Luehmann et al., 2003; Gouras et al., 2010), a large amount of the required Aβ peptides comes from a pool of soluble Aβ in the ISF (Cirrito et al., 2008; Funke, 2011).

To investigate the dynamics of soluble Aβ, hippocampal MD was used in awake transgenic mice before and during the process of Aβ plaque formation (Hong et al., 2011). They found that diffusible forms of Aβ, predominantly Aβ42, came from a large reservoir of less soluble Aβ42 in brain parenchyma and decreased in ISF during deposition of Aβ (Hong et al., 2011). Additional *in vitro* and *in vivo* MD experiments were able to demonstrate a linear correlation between neuronal activity and the interstitial Aβ concentrations (Kamenetz et al., 2003; Cirrito et al., 2005, 2008). Following TBI, decreased electroencephalographic (EEG) activity in the hippocampus occurred concomitantly with decreased MD hippocampal Aβ levels, supporting the hypothesis that a TBI-induced reduction in neuronal activity may lead to reduced ISF Aβ levels (Schwetye et al., 2010).

For human use, most MD studies evaluate either the 20 or the 100 kDA cut-off MD catheters (Hutchinson et al., 2005; Hillman et al., 2006). Since Aβ40 or Aβ42 peptides have a molecular weight (MW) of ∼4.5 kDa, both catheters could be used. However, if T-tau (*vide infra*) is also evaluated, the 100 kDa catheter needs to be used due to the 48–67 kDa MW of tau proteins (Ost et al., 2006). Cerebral MD has recently been used in humans with severe TBI for the study of interstitial Aβ changes (Brody et al., 2008; Marklund et al., 2009; Magnoni and Brody, 2010; Magnoni et al., 2012) (Table 4). In an early study, MD and an Aβ1-x ELISA was used to analyze every Aβ peptide species from amino acid 1–28 or higher (Brody et al., 2008). A key finding was that ISF Aβ peptides levels were lower than in ventricular CSF explained by a 30% MD recovery. When Aβ1-x levels were compared to Aβ40 and Aβ42 in pooled 8 h-samples, the latter were 2.5 and 35 times lower, respectively, suggesting that most Aβ peptides in the injured human brain are neither Aβ40 nor Aβ42. Finally, in most patients did the ISF Aβ levels increase over time and the level of consciousness correlated well with ISF Aβ levels, implying a link to synaptic activity (Brody et al., 2008). An additional study from the same group (Magnoni et al., 2012) showed that although the MD Aβ levels were lower when the MD catheter was placed in the pericontusional tissue compared to a non-contusional area, pericontusional Aβ levels increased more substantially over time. Another MD study analyzed ISF Aβ40 and Aβ42 levels in patients with severe TBI where higher Aβ42 levels were found in patients with diffuse TBI compared to focal TBI patients (Marklund et al., 2009). Notably, MD Aβ40 levels were above detection level in only half of the patients in this study (Marklund et al., 2009).

These studies indicated that MD is a useful tool to study Aβ dynamics in the injured brain following TBI. Given the lack of baseline, uninjured control Aβ values, alterations in the Aβ peptides levels following TBI should be interpreted with caution. It has been hypothesized that reduced Aβ production may be due to neuronal loss and/or decreased synaptic activity (Cirrito et al., 2005, 2008; Brody et al., 2008; Magnoni and Brody, 2010) and may be increased by axonal injury (Marklund et al., 2009). Although it has been speculated that toxic Aβ byproducts such as oligomers and protofibrils initiate cascades ultimately leading to neurodegeneration and dementia (Magnoni and Brody, 2010), available evidence is insufficient to imply a causative role for the early post-injury Aβ changes. Moreover, it should be stressed that brain ISF is not in full equilibrium with the CSF (Fishman, 1992; Brody et al., 2008) and the half life of Aβ in brain tissue has not been established. Larger patient series are needed to investigate their relationship with clinical outcome and discern possible differences between injured and uninjured brain regions as well as between focal and diffuse TBI.

## Tau as a Biomarker Following TBI

Total tau is present in abundance in the central nervous system and in particular in unmyelinated axons and cortical interneurons (Trojanowski et al., 1989; Sivanandam and Thakur, 2012). Its biological activity is regulated by phosphorylation (P-tau), which has been associated with various neuropathologies (Alonso et al., 2001; Feijoo et al., 2005; Morris et al., 2011). Following human TBI, C-tau is considered a reliable biomarker of neuronal injury (Shaw et al., 2002; Zemlan et al., 2002; Gabbita et al., 2005) and has been suggested to be an indicator of axonal injury (Trojanowski et al., 1989; Wilhelmsen, 1999; Zemlan et al., 1999; Emmerling et al., 2000; Franz et al., 2003; Ost et al., 2006; Zetterberg et al., 2006; Magnoni et al., 2012; Sivanandam and Thakur, 2012). NFTs are formed by abnormal, phosphorylated tau filaments and CSF tau are commonly increased 3–4 times in AD (Blennow and Hampel, 2003; Selkoe and Schenk, 2003; Sivanandam and Thakur, 2012). Tau levels can be markedly increased in the CSF after TBI (Table 3) and show promise also as a specific serum biomarker in the human (Liliang et al., 2010b) and experimental setting (Rostami et al., 2012).

**Figure 1:**
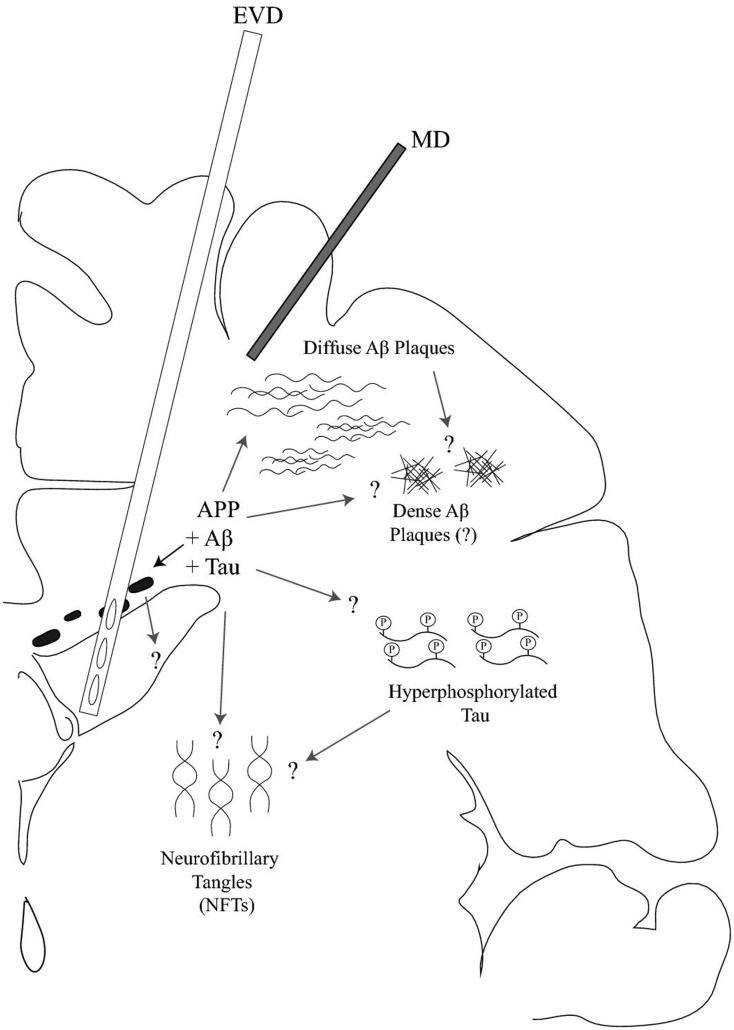
**Schematic drawing of interstitial fluid (ISF) and cerebrospinal fluid (CSF) sampling of tau protein and amyloid-β (Aβ) peptides following traumatic brain injury (TBI) on a coronal brain section**. An external ventricular drainage (EVD) and a microdialysis (MD) catheter are placed into the frontal horn of the ventricular system and superficial cortex, respectively. Initially, TBI results in an accumulation of amyloid precursor protein (APP) that, following its degradation, may lead to intra-axonal amyloid-β (Aβ) accumulation and plaque formation in the brain parenchyma. Following TBI, early Aβ plaques are typically of the diffuse type in contrast to those observed in Alzheimer’s disease whereas dense plaques may be observed in patients surviving for many years post-injury. Alternatively, Aβ peptides may also be produced by normal neuronal activity and be reduced by TBI. Neurofibrillary tangles (NFTs) can also be formed after TBI as a consequence of hyperphosphorylated tau. In humans, NFT formation does not appear to occur acutely and has mainly been observed beyond 4 weeks post-injury following a single, severe TBI. However, hyperphosphorylated tau aggregations can be observed as a characteristic observation following repetitive mild TBI. The question marks illustrate the unknown features of Aβ and tau accumulation, their release into the CSF or ISF, or the dynamic distribution between the CSF and ISF levels of Aβ and tau.

There is evidence to support that P-tau is important in the development of neurodegeneration (see previous section). Apoε deficiency and TBI have both been associated with hyperphosphorylation of a tau protein domain (Genis et al., 2000; Sivanandam and Thakur, 2012) (Figure 1). Additionally, tau misprocessing can be caused by abnormal accumulation of Aβ and tau *per se* may mediate Aβ cytotoxicity in AD (Le et al., 2012), adding to the complexity of tau and Aβ changes following TBI.

## Tau in CSF and ISF Following TBI

Previous studies have consistently shown that tau CSF levels, which have been closely linked with the presence of axonal injury, increased intracranial pressure, and clinical outcome, are increased in TBI patients compared to normal controls (Zemlan et al., 2002; Franz et al., 2003; Ost et al., 2006; Zetterberg et al., 2006; Liliang et al., 2010b; Magnoni et al., 2012) (Table 3). The results are different in milder forms of TBI, including boxing, since tau levels are only slightly increased or even unchanged (Zetterberg et al., 2006; Neselius et al., 2012). When evaluating tau as a biomarker following TBI, it must be considered that ventricular CSF typically has higher tau levels than lumbar CSF (Blennow and Nellgard, 2004).

Only recently has tau also been analyzed in the ISF (Table 4). Using MD, ISF T-tau levels were clearly above the detection limit in all patients and were higher in patients with a focal/mixed TBI compared to DAI patients (Marklund et al., 2009). The ISF tau levels were comparable to those previously measured in ventricular CSF post-TBI (Franz et al., 2003; Ost et al., 2006). Recently, MD tau levels were found to be markedly higher in TBI patients with the MD probe placed in the pericontusional area compared to when the MD probe was placed in a brain region without contusions. Additionally, high initial ISF tau levels correlated with poor clinical outcome (Magnoni et al., 2012). The MD recovery of tau is likely low, estimated to be 1–2% (Magnoni et al., 2012), since hyperphosphorylation markedly decreases the solubility of tau (Table 4). Although T-tau has commonly been analyzed as biomarkers, the phosphorylation status of tau is likely more important in the pathophysiology of TBI to date.

## Conclusion and Future Directions

The current literature on early and late CSF, ISF, and brain tissue changes of Aβ peptide levels and tau following TBI was reviewed. To define the precise relation between Aβ and tau levels in brain tissue, CSF and/or blood and clinical disease remains an important scientific challenge due to the association between TBI and the risk of developing neurodegeneration and AD. Available experimental and clinical evidence implies a complex relationship between increased tau protein release, Aβ peptide deposition, and NFT and Aβ plaque formation following TBI. Rodent studies, perhaps most importantly those carried out in transgenic mice, have provided important mechanistic information and shed light into many aspects of tau and Aβ formation following TBI although without consistently mimicking the histopathological findings observed in humans. TBI severity, the used species and model, choice of analytical technique, and the inherent difference between human and rodent brain may contribute to the inconsistent results obtained using experimental TBI models. On the other hand, the swine TBI model appears to produce Aβ pathology more closely resembling the human situation. Only biomarker analysis of Aβ peptides and tau may not be sufficient to elucidate the complex cellular, biochemical, genetic (e.g., neprilysin and Apoε4), and metabolic cascades ultimately predisposing TBI victims to an increased risk for AD. It appears likely that TBI accelerates the process leading to AD, although the mechanisms and relation to the acute injury cascade remain largely unknown. Possibly, many additional *in vitro* and *in vivo* experiments dissecting various aspects of the tau/Aβ cascade are needed. It is expected that the increased use of tau and Aβ peptides as biomarkers in the clinical setting will enhance our understanding of the link between TBI and the later development of AD.

Available studies show that Aβ and tau can be analyzed in interstitial and CSF although the analysis methods and the resulting biomarkers levels differ markedly among studies. The studies are mainly observational and long-term follow up data is frequently lacking. However, robust data exist for tau, showing elevated levels in the CSF and the ISF and a correlation between tau levels in both compartments and long-term outcome was also suggested. Emerging data suggest that tau is promising as a biomarker also in peripheral blood. The interpretation of post-injury Aβ levels is currently more complicated. Aβ peptides are produced both by normal neuronal metabolism and by enzymatic processing of accumulated APP in injured axons following TBI. Thus, their levels may be related to the level of consciousness, the presence of axonal injury or both and be reduced in the vicinity of cortical contusions. Importantly, increased Aβ peptide levels, particularly the longer and fibrillary ones, can also be neurotoxic *per se* (Brody et al., 2008; Marklund et al., 2009; Magnoni et al., 2012). Different analysis methods also render comparisons between studies difficult. Although the Aβ1-42 peptide is important in AD and has attracted much interest in TBI, other subspecies may also be highly relevant and much recent interest is directed toward Aβ oligomers and protofibrils (Magnoni and Brody, 2010).

Then, what is the current and future potential of tau protein and Aβ peptides as biomarkers and what can they tell us about the possible neurodegeneration occurring post-TBI? Ideally, the levels of a biomarker should closely correlate with a biological or pathogenic process (Czeiter et al., 2012) or be used as surrogate end-points. Obviously, the chronic sequelae of TBI survivors are crucial. However, at the current level of knowledge, the correlation between early Aβ and tau biomarker findings and the later development of AD is weak. Interestingly, it has been shown that acute Aβ accumulations can be reversed following TBI (Smith et al., 1998). Moreover, the vast complexity and variability in the used TBI models do not allow clear conclusions or extrapolation of the experimental results into clinical practice to date. Instead, available evidence suggests that Aβ and tau could be used as injury markers or in mechanistic studies. In future studies, correlation of levels in ISF, CSF, and/or serum with advanced neuroimaging such as diffuse tensor imaging or Positron Emission Tomography (PET) using, e.g., Pittsburgh Compound B (Quigley et al., 2011) preferably using rapid biomarker sampling combined with enhanced analytical tools could provide additional information. Long-term and serial biomarker determination would also be of importance where potential differences in the biomarker levels in lumbar versus ventricular CSF could be evaluated. BACE1 inhibitors, γ-secretase inhibitors, statins, and neprilysin replacement therapy are emerging treatment possibilities for AD which could also play key roles in the future study of TBI. Combined with biomarker analysis, these pharmacological tools could provide crucial information related to the importance of tau and Aβ peptides in the pathophysiology and long-term consequences of TBI.

## Conflict of Interest Statement

The authors declare that the research was conducted in the absence of any commercial or financial relationships that could be construed as a potential conflict of interest.
